# Chloroquine enhanced the anticancer capacity of VNP20009 by inhibiting autophagy

**DOI:** 10.1038/srep29774

**Published:** 2016-07-14

**Authors:** Xiaoxin Zhang, Qiaoqiao Xu, Zhuangzhuang Zhang, Wei Cheng, Wenmin Cao, Chizhou Jiang, Chao Han, Jiahuang Li, Zichun Hua

**Affiliations:** 1 The State Key Laboratory of Pharmaceutical Biotechnology, School of Life Science and School of Stomatology, Affiliated Stomatological Hospital, Nanjing University, Nanjing, 210093, Jiangsu, China; 2 Changzhou High-Tech Research Institute of Nanjing University and Targetpharma Laboratory, Changzhou 213164, Jiangsu, China; 3 College of Pharmacy, Nanjing University of Chinese Medicine, Nanjing 210046, China; 4 The State Key Laboratory of Bioelectronics, Southeast University, Nanjing 210018, China

## Abstract

Bacteria-based living anticancer agents have emerged as promising therapeutics. However, the functional role of autophagy in bacterial cancer therapy has been little investigated. In this study, *Salmonella* VNP20009 induced autophagy in B16F10 cells, which is an unfavorable factor in bacterial cancer therapy. Inhibiting the induction of autophagy by chloroquine or siRNA in bacterial cancer therapy dose- and time-dependently promoted cell death. The combined therapy of VNP20009 and chloroquine not only enhanced the bacterial tumor targeting ability but also facilitated the infiltration of immune cells into the tumor. Our results showed that the combined therapy of VNP20009 and chloroquine could significantly inhibit tumor growth and prolong mouse survival time. This study provides a novel strategy for improving the anti-cancer efficacy of bacterial cancer therapy.

In the late 19^th^ century, Dr. William Coley first discovered that bacteria can be employed to cure cancer. Over the subsequent 200 years, several bacteria have been studied for bacterial cancer therapy, and among them *Salmonela* has been investigated the most^1^. *Salmonella* can preferably accumulate in tumor and inhibit tumor growth^2^,^3^,^4^. However, wild type *Salmonella* is a pathogen and causes a severe infection in humans. To address this issue, VNP20009 was developed by depleting *msbB* and *purI* in the background of *Salmonella* 14028 and was widely used to carry plasmids and shRNA to treat tumor-bearing mice^4^,^5^,^6^,^7^,^8^,^9^. Although VNP20009 can specifically accumulate in tumor and inhibit tumor growth in a murine model, it failed in a phase I clinical trial because few bacteria were found in the tumors of patients infected with VNP20009^10^. Thus, the mechanism through which bacteria interact with the host must be elucidated to improve its tumor targeting ability and anti-cancer capacity.

Macroautophagy (autophagy) is a highly conserved catabolic process that involves the formation of double-membrane vesicles, which sequester cytosolic components and deliver them to the lysosome for degradation^11^. Numerous studies have shown that autophagy is an important host defense pathway because it restricts intracellular replication of bacteria^12^,^13^. *Salmonella* can considerably induce autophagy, which protects against *Salmonella* infection^14^,^15^,^16^. Although autophagy performs a cytoprotective role in a bacterial infection, it serves as a flexible role in cancer cells^17^. Under normal conditions, autophagy can remove unfolded proteins, damaged organs as well as instable genomes to prevent cancer development. By contrast, high levels of autophagy are induced in tumor cells during cancer development to allow survival in hypoxic, nutrient-limited and acidosis environments. Because autophagy can either inhibit or promote cancer cell proliferation, using autophagy for therapeutic purposes in cancer treatment requires a careful consideration.

In this study, we provide evidence that VNP20009 induced autophagy in B16F10 cells, and we elucidated the functional role of autophagy in bacterial cancer therapy. B16F10 murine melanoma cells are widely used in bacterial cancer therapy because they are highly aggressive and low immunogenic^18^,^19^,^20^. Previous studies showed that *Salmonella* could significantly inhibit B16F10 tumor growth and prolong mouse survival time. The results showed that inhibiting autophagy induction upon bacterial cancer therapy could enhance tumor cell death, bacterial tumor targeting ability and the ability of immune system to fight cancer. The combined therapy of VNP20009 and chloroquine could significantly inhibit tumor growth and prolong mouse survival time. This study reveals the key role of autophagy in bacterial cancer therapy.

## Results

### VNP20009 induces autophagy in B16F10 murine melanoma cells

**Figure 1:**
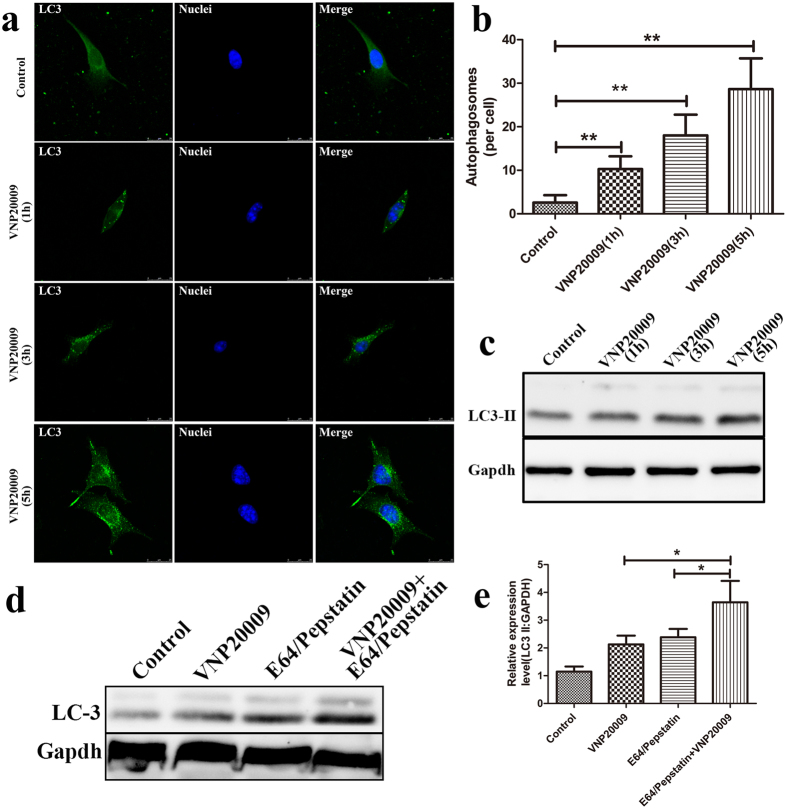
VNP20009 induced autophagy in B16F10 murine melanoma cells. (**a**) B16F10 cells transfected with GFP-LC3 were infected with VNP20009 and fixed at indicated times post transfection. Cells were then stained with DAPI to determine nucleus. Cells were analyzed by fluorescence microscopy. (**b**) The formation of vacuoles containing GFP-LC3 per cell was analyzed by Image-Pro Plus. Data are presented as mean ± SD. **p < 0.01, as compared with untreated group. (**c**) B16F10 cells were infected as in A. Immunoblot analyses for LC3-II. (**d**) B16F10 cells were infected by VNP20009 for one hour. Immunoblot analyses for LC3-II in the presence or absence of E64d and pepstatin A. (**e**) Protein expression of LC3-II were analyzed by ImageJ. Data are presented as mean ± SD. *p < 0.05.

An increasing number of studies have shown that autophagy can target *Salmonella* in damaged *Salmonella*-containing vacuoles or cytosol and thus protect against *Salmonella* infection. In this study, we aimed to investigate the functional role of autophagy induced by VNP20009 in B16F10 murine melanoma cells. B16F10 cells transfected with GFP-LC3 were infected with VNP20009 at the multiplicity of infection (MOI) of 1:1 for one hour. To reduce the cytotoxicity of VNP20009, gentamicin was added to the plates to kill extracellular bacteria one hour after infection. As shown in Fig. 1a, the green fluorescence was weak and diffuse in the cytoplasm of the control group. By contrast, punctate fluorescence was significant in the VNP20009-treated group and increased upon VNP20009 treatment in a time-dependent manner (Fig. 1a). The number of punctate GFP-LC3 per cell in the VNP20009 treated group at indicated times after infection (i.e., 1, 3 and 5h) was 5-, 9- and 15-fold higher than that in the VNP20009 treated group, respectively (Fig. 1b).

Western blot analysis was also performed to analyze the production of LC3-II, a cleaved LC3-phosphatiyl-ethanolamine conjugate and a general marker of autophagy. As predicted, VNP20009 treatment significantly enhanced the formation of LC3-II, which was further accumulated with prolonged VNP20009 treatment (Fig. 1c). Similar results were observed in human melanoma cell line (A375). As shown in Supplementary Figure S1, the average amount of GFP-LC3 dots was significantly increased upon VNP20009 treatment in a time dependent manner. Western blot analysis also confirmed the enhanced autophagic activity, as LC-3 II levels increased in the VNP20009 treated group in a time-dependent manner.

To further confirm the activation of autophagy after VNP20009 infection, we measured the autophagic flux in the presence or absence of lysosomal proteases inhibitor E64d and pepstatin A, which are widely used to examine the transit of LC-3 II through autophagic flux. Compared with VNP20009 treatment alone, the amount of LC-3 II was higher in the presence of the inhibitors (Fig. 1d,e). These results suggested that VNP20009 induced autophagy in B16F10 murine melanoma cells.

### Inhibition of autophagy induced by VNP20009 induces cell death more potently

**Figure 2:**
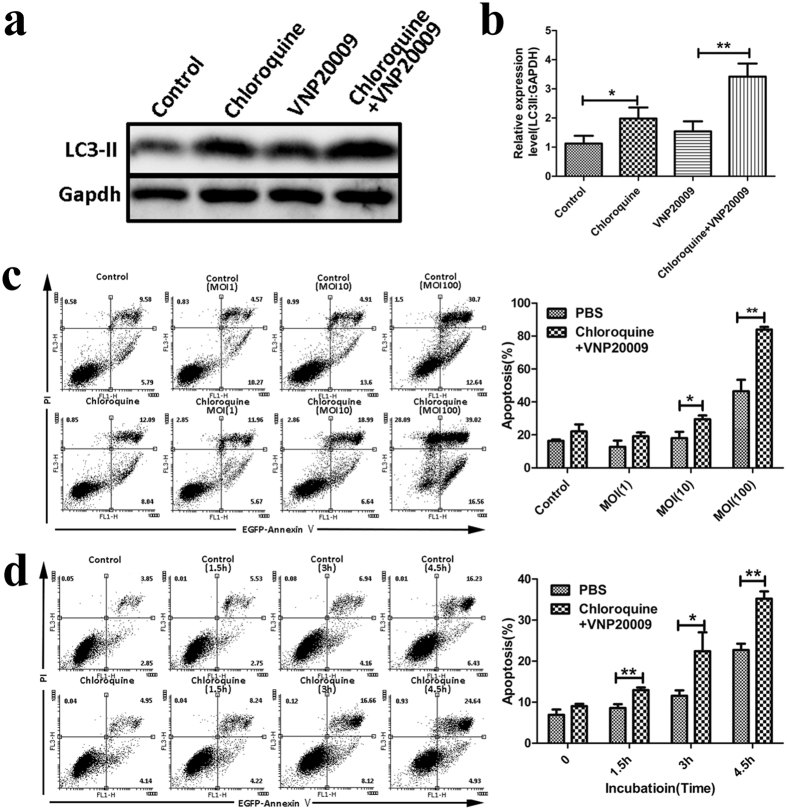
Inhibition of autophagy by chloroquine induced more cell death than VNP20009 treatment. (**a**) B16F10 cells were infected with VNP20009 in the presence or absence of 20 μM chloroquine. Immunoblots showed the expression of LC3-II. (**b**) Protein expression of LC3-II was analyzed by ImageJ. Data are presented as mean ± SD. *p < 0.05, **p < 0.01. (**c**) B16F10 cells were infected with VNP20009 at different MOI in the presence or absence of 20 μM chloroquine. Cell death was analyzed using flow cytometry. Data are presented as mean ± SD. *p < 0.05, **p < 0.01. (**d**) B16F10 cells were infected with VNP20009 at MOI of 10:1 in the presence or absence of 20 μM chloroquine for indicated time. Cell death was analyzed using flow cytometry. Data are presented as mean ± SD. *p < 0.05, **p < 0.01.

Although VNP20009 induced autophagy in B16F10 cells and this process is important for restricting bacterial growth, it remains unclear if inhibiting autophagy is toxic to VNP20009-infected B16F10 cells. In this regard, we first inhibited autophagy by chloroquine, a common autophagy inhibitor. As shown in Fig. 2a, chloroquine induced an increase in LC3-II as it raises the lysosomal pH and inhibits both fusion of autophagosome with lysosome and lysosomal protein degradation. Although the LC3-II level increased after VNP20009 treatment, the LC3-II levels were most prominent after the combined treatment of chloroquine and VNP20009, as indicated by the strong LC3-II band on the Western blots (Fig. 2a). The expression of LC3-II in the combined treatment was a two-fold increase compared to that in the chloroquine or VNP20009 treatment alone (Fig. 2b). Hence, VNP20009 induced autophagic flux, which could be inhibited by chloroquine.

After confirming that chloroquine could inhibit the autophagic flux induced by VNP20009, we pretreated B16F10 cells with chloroquine for 6 h and then added VNP20009. An annexin V/PI assay indicated that although chloroquine did not affect cell viability, VNP20009 lead B16F10 cell death in a time- and dose-dependent manner after pre-inhibition of autophagy (Fig. 2c,d). Cell death was enhanced with prolonged VNP20009 treatment. However, the combined treatment significantly induced more cell death than VNP20009 treatment alone at all assessed time points (Fig. 2d). During treatment with different MOI for the same duration, cell death increased with increasing MOI (Fig. 2c).

**Figure 3:**
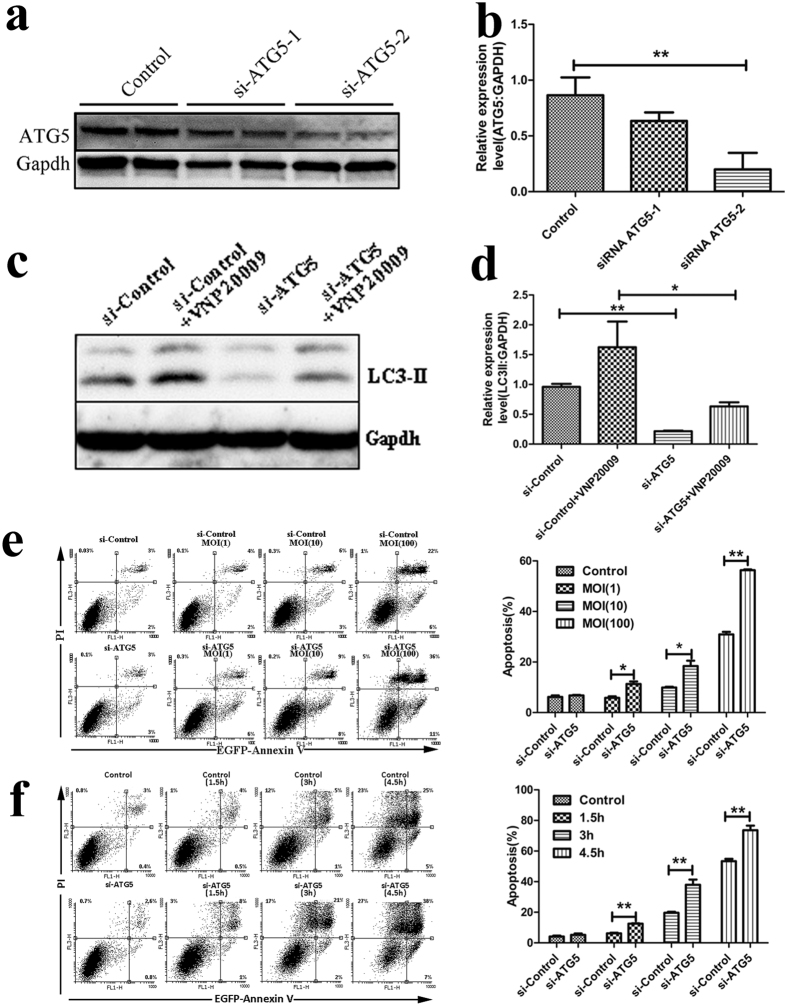
Inhibition of autophagy by siRNA induced more cell death than VNP20009 treatment. (**a**) Western blots of ATG5 protein expression from B16F10 lysates after transfection of siRNA with potential for silencing ATG5. (**b**) Protein expression of ATG5 was analyzed by ImageJ. Data are presented as mean ± SD. **p < 0.01. (**c**) Protein expression of LC3-II after infected with VNP20009 in the wild type or ATG5 knock out cells. (**d**) Protein expression of LC3-II was analyzed by ImageJ. Data are presented as mean ± SD. *p < 0.05, **p < 0.01. (**e**) The wild type or ATG5 knock out cells were infected with VNP20009 at different MOI. Cell death was analyzed using flow cytometry. Data are presented as mean ± SD. *p < 0.05, **p < 0.01. (**f**) The wild type or ATG5 knock out cells were infected with VNP20009 at MOI of 10:1 at indicated time. Cell death was analyzed using flow cytometry. Data are presented as mean ± SD. *p < 0.05, **p < 0.01.

Autophagy-related gene 5 (ATG5) is essential for vesicle elongation during autophagy. To confirm that inhibiting autophagy in VNP20009-treated B16F10 cells induced cell death, we silenced ATG5 by siRNA. As shown in Fig. 3a,b, siRNA-ATG5-2 significantly silenced the expression of ATG5 (75%) compared with that in the control. After silencing ATG5, autophagic flux in VNP20009 treatment was significantly reduced, as indicated by decreased intensity of the LC3-II band on the western blots (Fig. 3c,d). VNP20009 was then added to ATG5 wild-type and ATG5 deficient cells to determine whether VNP20009-induced autophagy acts as a pro-survival mechanism in bacterial cancer therapy. As shown in Fig. 3e,f, VNP20009 treatment alone triggered cell death in a time- and dose-dependent manner. However, the incidence of cell death was higher in the ATG5 siRNA group than that in the control group at all assessed time points (Fig. 3f). Moreover, treatment with different MOI for the same duration significantly induced higher cell death rates in the ATG5 siRNA group than that in the control group (Fig. 3e). Inhibition of autophagy by ATG7 siRNA also increased the sensitivity of B16F10 cells to VNP20009 (Supplementary Fig. S2). These results suggested that VNP20009-induced autophagy acts as a pro-survival mechanism in B16F10 murine melanoma cells.

### *In vivo* inhibition of autophagy potentiates the anticancer capacity of VNP20009

**Figure 4:**
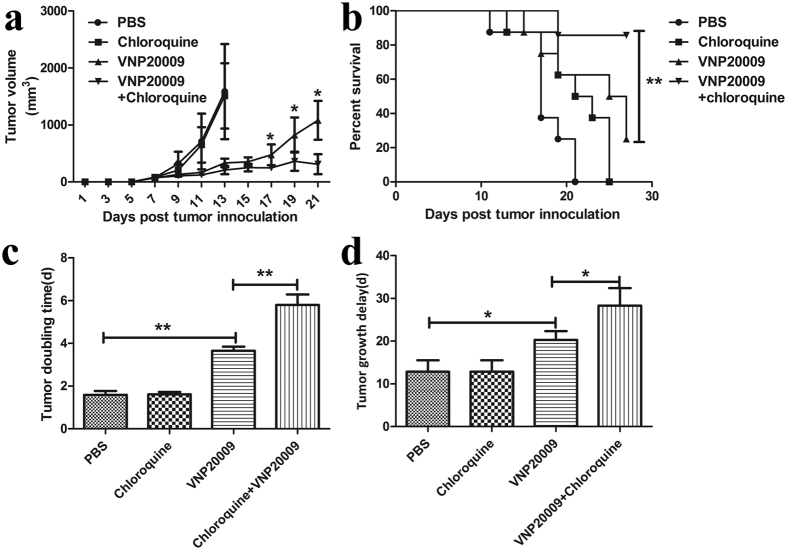
Synergistic inhibition of tumor growth in mice treated with VNP20009 and chloroquine. (**a**) Tumor bearing mice (n = 8, each group) were treated with PBS, chloroquine, VNP20009 and VNP20009 plus chloroquine. Tumor volumes among different groups were compared. Data are presented as mean ± SD. *P < 0.05. (**b**) Kaplan-Meier survival curves of mice bearing B16F10 melanomas. Data were analyzed by the log-rank test. **P < 0.01. (**c**) Tumor doubling time. Data are presented as mean ± SD. **P < 0.01. (**d**) Tumor growth delay. Data are presented as mean ± SD. *P < 0.05.

Considering that VNP20009 induces more cell death after inhibiting autophagy, we assessed the potential of the combined therapy of VNP20009 and chloroquine *in vivo*. When the tumor was palpable, the animals were treated with: (1) PBS, (2) VNP20009, (3) chloroquine, and (4) chloroquine plus VNP20009. The amount of chloroquine used in this study was 60 mg/kg, which is similar to the dose employed by Patrizia Agostinis^21^. As shown in Fig. 4a, chloroquine treatment alone did not significantly affect tumor size, consistent with the findings of Agostinis^21^. However, the combined treatment of VNP20009 and chloroquine significantly inhibited tumor growth and prolonged mouse survival time (Fig. 4a,b). As shown in Fig. 4c,d, the tumor doubling time significantly increased from 3.65 d (CI, 3.47–3.85 days) in the VNP20009 group to 5.80 d (CI, 5.33–6.3 days) in the VNP20009 plus chloroquine group (p < 0.05). The tumor growth delay significantly increased from 20.27 d (CI, 18.23–22.38 days) in the VNP20009 group to 28.29 d (CI, 24.29–32.52) in the VNP20009 plus chloroquine group (p < 0.05). The results indicated that chloroquine treatment could enhance the anticancer capacity of VNP20009.

### The combined therapy of VNP20009 and chloroquine enhances tumor cell death, bacterial tumor targeting ability and the ability of immune system to fight cancer

**Figure 5:**
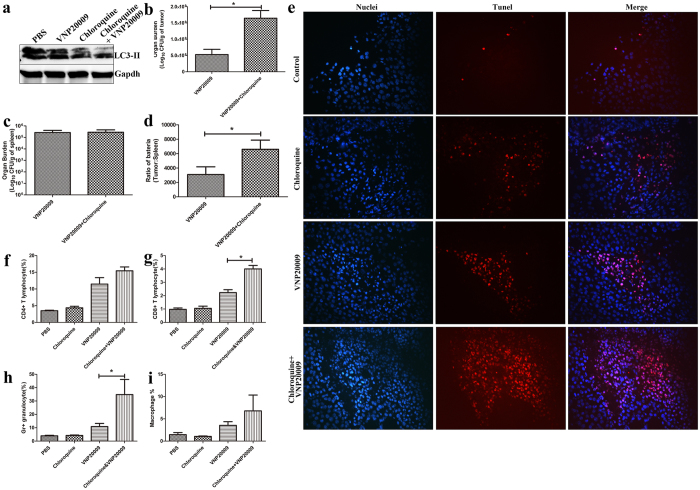
The combined therapy of VNP20009 and chloroquine enhanced bacterial tumor targeting ability, cytotoxic effects and immune responses against cancer cell. (**a**) Immunoblots showed the expression of LC3-II. Tumor bearing mice were treated as mentioned in material and methods and sacrificed on the 7^th^ day post bacterial inoculation. Tumor lysates were analyzed by western blot. (**b**) VNP20009 were i.p. inoculated into the tumor-bearing mice (n = 6, in each group). The bacterial titer in tumor was calculated, *P < 0.05. (**c**) VNP20009 were i.p. inoculated into the tumor-bearing mice (n = 6, in each group). The bacterial titer in spleen was calculated. (**d**) Tumor/spleen ratio of tumor bearing mice treated with the combined therapy of VNP20009 plus chloroquine or VNP20009. *P < 0.05. (**e**) TUNEL staining showed the death of B16F10 melanoma cells from the mice treated with PBS, chloroquine, VNP20009 and VNP20009 plus chloroquine. (**f**–**i**) Tumor bearing mice (n = 4, per group) were treated as mentioned in material and methods. Tumors were excised on the 7^th^ day post bacterial innoculation and single-cell suspensions were generated. f, Percentage of CD4+ T cell. g, Percentage of CD8+ T cell. h, Percentage of granulocyte. i, Percentage of macrophage. Data are presented as mean ± SD. *p < 0.05.

After confirming that chloroquine treatment inhibited autophagy *in vivo*, we counted the number of bacteria in tumor and spleen, where bacteria usually accumulate (Fig. 5a). Three days post-infection, more bacteria targeted tumor after combined therapy. The bacteria titer of the combined therapy exhibited a three-fold increase compared to the VNP20009 treatment alone group (Fig. 5b). However, the bacteria titer of the combined therapy in spleen was not significantly different from that of the VNP20009 treatment (Fig. 5c). Additional calculations were performed to compare the ratio between tumor and spleen bacterial loads. The ratio of combined therapy is two times greater than that of VNP20009 treatment (Fig. 5d).

Next, we stained the tumor sections with an *in situ* TUNEL assay to determine whether the synergistic effects of the combined therapy on tumor size reflect enhanced death of melanoma cells. Similar to the results *in vitro*, the combined treatment exhibited a strong pro-death effect *in vivo*, as indicated by nearly all areas staining positively for TUNEL (Fig. 5e). By contrast, less cell death was observed in the VNP20009 and chloroquine group. Hence, the combined treatment of VNP20009 and chloroquine could inhibit tumor growth by inhibiting VNP20009-induced protective autophagy and promoting cell death.

Accumulating studies highlight that autophagy affects anti-tumor immunity^22^. To delineate the role of autophagy in immunosuppression within tumor, we analyzed the immune cells in tumor by flow cytometry. Consistent with our previous findings, VNP20009 treatment significantly increased the number of CD4^+^, CD8^+^, F4/80^+^ and Gr-1^+^ cells in the tumor (Fig. 5f–i). The combination of VNP20009 and chloroquine enhanced this effect. The number of CD8^+^ and Gr-1^+^ cells was significantly increased in the combined treatment. There was a two-fold increase in the number of CD8^+^ T cells in the tumors of mice treated with the combined therapy than the mice treated with VNP20009 (Fig. 5g). The number of Gr-1^+^ cells in tumors increased from 10% in the VNP20009 group to 34% in the combined therapy group (Fig. 5h).

## Discussion

In this study, we provide the initial evidence that autophagy could not only be induced by VNP20009 in B16F10 cells, but could also counteract the anticancer capacity of VNP20009. The inhibition of autophagy induction in bacterial cancer therapy could enhance cancer cell apoptosis and bacterial tumor targeting ability as well as the infiltration of immune cells into tumor. Our results showed that the combined therapy of VNP20009 and chloroquine could significantly inhibit tumor growth and prolong mouse survival time when compared with VNP20009 treatment alone.

Several studies have suggested that autophagy is an innate immune response and can target intracellular bacteria in the cytosol, damaged vacuoles and phagosomes to inhibit bacterial growth^13^,^23^. When *Salmonella* enters the cell, they are restricted to reside in *Salmonella*-containing vacuole (SCV). However, during early infection, a population of bacteria damages the SCV membrane by SPI-1 and escapes to the cytosol to obtain nutrients for rapid growth^12^. Autophagy can target *Salmonella* in the cytosol by ubiquitin and sugar signals to protect bacterial replication in cytosol^14^,^24^. The current study revealed that VNP20009 enhanced the formation of LC3-II, indicating that these processes were targeted by autophagy in B16F10 cells, which was in consistent with the recent work of Lijun Jia^25^. Although their study demonstrated that inhibiting autophagy could increase intracellular bacteria multiplication and bacteria-mediated cancer cell killing *in vitro*, a further *in vivo* experiment was lacking, which was conducted in this study. Our results showed that more bacteria accumulated in the tumors of mice after inhibiting autophagy in the VNP20009 treated group *in vivo*. Taken together, uncontrolled bacterial replication is a likely candidate factor for the enhanced therapeutic effects of the combined treatment.

Chloroquine, the treatment for rheumatoid arthritis, SLE, HIV and malaria for half a century, is one of the only autophagy inhibitors, whose effectiveness and safety have been proven in clinical trials and have been approved by the FDA^26^. Chloroquine is a weak base and accumulates in lysosomes, leading to its alkalinization. Thus, it prevents autophagosome-lysosome fusion and the degradation of autophagosomes. Accumulating evidence showed that chloroquine can be applied alone in Myc-driven lymphoma, pancreatic cancers and metastatic mammary carcinoma to inhibit cell proliferation, increase cell apoptosis, or normalize tumor vessel or chloroquine can be used in combination with other therapies^21^,^27^,^28^,^29^,^30^. When combined with chemotherapy agents, chloroquine enhanced the cytotoxic effects of chemotherapy and induced cell apoptosis by increasing the levels of intracellular misfolded proteins^31^,^32^. In the present study, the combination of VNP20009 and chloroquine induced cell death in a dose- and time-dependent manner, which is dependent on autophagy. Considering that autophagy is generally believed to confer a survival advantage for tumor cells, we suggested that inhibiting autophagy could enhance the cytotoxicity of VNP20009.

When applied in cytokine therapy, chloroquine would enhance immunotherapeutic efficacy and limit toxicity^33^. Autophagy has been reported to play a “decisive” role in the cancer immune microenvironment, and the role of autophagy in antitumor immunity is context dependent. In the present study, we showed that more immune cells infiltrated into the tumors of mice treated with the combined therapy of VNP20009 and chloroquine. In an established tumor, where the cells are deprived of oxygen and nutrients, autophagy is induced to promote tumor cell resistance to the activity of cytotoxic T cells and natural killer cells. Blocking autophagy can enhance immune responses to tumor^34^,^35^. VNP20009 is also an immune activating agent that exerts anti-tumor effects by inhibiting tumor indoleamine 2, 3-dioxygenase 1 expression, and inducing gap junctions in tumors and Th1-type cellular immune responses^18^,^19^,^20^,^36^. The results showed that inhibiting autophagy induction following bacterial infection could increase the capacity of VNP20009 to boost the immune system to fight against cancer.

In this study, we showed that autophagy in bacterial cancer therapy has adverse effects on limiting bacterial replication in tumors, contributing to tumor cell’s resistance to the cytotoxicity of VNP20009 and inhibiting the immune responses to the tumor. The unique combination of VNP20009 and chloroquine can enhance the anticancer activity of VNP20009 by inhibiting tumor growth more potently and prolonging mouse survival time. This study provides a novel perspective of autophagy in bacterial cancer therapy and potentiates future studies and applications of combined therapies.

## Methods

### Bacteria, cell lines and animals

Lipid A modified (msbB-), auxotrophic (purI-) *Salmonella typhimurium* VNP20009 was obtained from American Type Culture Collection (ATCC) and cultured in modified Luria–Bertani (LB) medium. The B16F10 cells were obtained from ATCC and cultured in 5% CO_2_ in a humidified atmosphere in *Dulbecco*’*s Modified Eagle*’*s medium* (*DMEM*) with 10% fetal bovine serum (FBS). Six-week-old female C57/B6 mice were purchased from the Comparative Medicine Center of Yangzhou University and maintained in pathogen-free conditions for one week before the start of the experiment. The research protocols were approved by the Committee on the Use of Live Animals at Nanjing University and were performed in accordance with the approved guidelines.

### Chemicals and antibodies

Chloroquine (C6628) was purchased from Sigma Aldrich. E64 (S7379) was purchased from Selleckchem. Pepstatin (M183) was purchased from AMRESCO. Annexin-V-FITC was synthesized by our laboratory. Propidium iodide (PI) was purchased from Sunshine Biotechnology. Lipofectamine 2000 was purchased from Invitrogen. The antibody used were as follows. Anti-LC3-II (2775) was purchased from Cell Signaling Technology. Gapdh (D3015) was purchased from Santa Cruz Biotechnology. Anti-CD4-PE (557308) and Anti-Gr-FITC (551461) were purchased from BD. Anti-CD8-APC (E07056-1633) and anti-F4/80-FITC (E00611-1636) were purchased from eBioscience.

### Immunofluorescence Microscopy

The day before the experiment, B16F10 cells were transfected with GFP-LC3 expression plasmid using Lipofectamine 2000. B16F10 cells transfected with GFP-LC3 were infected with VNP20009 at MOI of 1:1 for one hour. Gentamicin (100 μg/ml) was added to the plates to kill extracellular bacteria one hour after infection. The gentamicin concentration was reduced to 10 μg/ml two hours later. The formation of vacuoles containing GFP-LC3 (dots) were visualized by fluorescence microscopy (TCS SP8, Leica) and counted as previously described using Image-Pro Plus^37^,^38^.

### Apoptosis assay

Cells, which were either treated with 20 μM chloroquine for 6 hours or the expression of ATG5 was silenced by siRNA, were incubated with VNP20009 at the indicated time and then stained with EGFP-Annexin V and propidium iodide to measure the rate of apoptosis by flow cytometry. Three independent experiments were performed, and each experiment was repeated three times.

### TUNEL staining of tumor tissue sections

Tumor tissue sections (10 μm) were prepared according to standard protocols and stained with TUNEL BrightRed Apoptosis Detection Kit (Vazyme). The images were obtained using Zeiss Axioplan 2. The number of TUNEL positive cells was quantified by Image-Pro Plus.

### Western blot

Proteins, which were extracted in lysis buffer and separated by SDS-PAGE, were electrophoretically transferred onto polyvinylidene fluoride membranes. The membranes were probed with antibodies overnight at 4 °C and incubated with a horse radish peroxidase-coupled secondary antibody. Detection was performed using Tanon 1600 and band intensity was quantified by Image J.

### Animal assay

For this assay, 1 × 10^5^ B16F10 cells were subcutaneously inoculated into mice. When the tumor size reached 100 mm^3^ (7–8 days), mice were randomly divided into 4 groups (8 mice per group). Mice in the PBS group were intraperitoneally (i.p.) injected with 100 μl of PBS. Mice in the VNP20009 and VNP20009 plus chloroquine groups were i.p. injected with 1 × 10^5^ colony forming unit (CFU) of VNP20009. On the 7^th^ day post tumor implantation, mice in the chloroquine and VNP20009 plus chloroquine group were i.p. injected with 60 mg/kg chloroquine every the other day. The length and width of the tumor were measured every two days using a Vernier caliper (Mytutoyo Co., Japan) across its two perpendicular diameters. Tumor volume was calculated using the following formula: tumor volume = length × width^2^ × 0.52. The number and dates of deaths of mice were recorded to calculate the survival rate.

### Bacterial biodistribution in tumor-bearing mice

To determine bacterial biodistribution, mice (6 mice per group) in the PBS, VNP20009, chloroquine and VNP20009 plus chloroquine groups were sacrificed on the 3^rd^ day post bacteria injection. The tissue samples (spleen and tumor) were weighed and homogenized in 2 ml of PBS and plated on LB agar by a serial dilution. After 16 hours of culture at 37 °C, the titer of bacteria was determined by counting colonies and dividing them by the weight of the tissue (CFU/g tissue).

### Flow cytometry

The tumor-bearing mice, which consist of 4 mice per group, were treated as described above and sacrificed on the 7^th^ day post bacterial injection. According to Vonderheide’ work, the tumors were collected post infection for subpopulation analysis of immune cells^39^. The tumor was digested by collagenase IV. A single cell suspension was obtained using a 40-μm filter to exclude red blood cells, which was then washed twice in DMEM medium. To determine the percentage of immune cells in the tumor, 10,000 cells suspended in 100 μl of DMEM containing 10% fetal calf serum (FCS) were stained for 30 min at 4 °C using the appropriate isotype controls, APC-conjugated anti-CD8, FITC-conjugated anti-Gr, PE-conjugated anti-CD4 and FITC-conjugated anti-F4/80. After washing in PBS, the cells were analyzed on a FACSCalibur using CELLQUEST software (BD Biosciences).

### Statistical analysis

Data are presented as the mean ± SD. Student’s t-test was used for the statistical analysis of the data. All statistical analyses were conducted using SPSS 10.0 statistical software (SPSS). P values of <0.05 or <0.01 were considered statistically significant.

## Additional Information

**How to cite this article**: Zhang, X. *et al*. Chloroquine enhanced the anticancer capacity of VNP20009 by inhibiting autophagy. *Sci. Rep.* **6**, 29774; doi: 10.1038/srep29774 (2016).

## Supplementary Material

Supplementary Information
