# Corrigendum: Chloroquine enhanced the anticancer capacity of VNP20009 by inhibiting autophagy

**DOI:** 10.1038/srep32468

**Published:** 2016-09-02

**Authors:** Xiaoxin Zhang, Qiaoqiao Xu, Zhuangzhuang Zhang, Wei Cheng, Wenmin Cao, Chizhou Jiang, Chao Han, Jiahuang Li, Zichun Hua

Scientific Reports 6: Article number: 2977410.1038/srep29774; published online: 07 14 2016; updated: 09 02 2016

The Acknowledgements section in this Article is incomplete.

“The authors are grateful to grants from the Doctoral Station Science Foundation from the Chinese Ministry of Education (20130091130003), the Nature Science Foundation of China (81421091), the Jiangsu Provincial Nature Science Foundation (BE2013630), the Open Project Programs from State Key Laboratory of Natural Medicines of China Pharmaceutical University (G140014) and The State Key Laboratory of Bioelectronics of Southeast University, the Bureau of Science and Technology of Changzhou (CZ20130011, CE20135013)”.

should read:

“The authors are grateful to grants from the Doctoral Station Science Foundation from the Chinese Ministry of Education (20130091130003), the Nature Science Foundation of China (81421091, 81573338), the Jiangsu Provincial Nature Science Foundation (BE2013630), the Open Project Programs from State Key Laboratory of Natural Medicines of China Pharmaceutical University (G140014) and The State Key Laboratory of Bioelectronics of Southeast University, the Bureau of Science and Technology of Changzhou (CZ20130011, CE20135013)”.

