# Supplementary material for: Chloroquine enhanced the anticancer capacity of VNP20009 by inhibiting autophagy
Source: Sci Rep. 2016 Jul 14;6:29774. doi: 10.1038/srep29774 (PMC4944202; doi:10.1038/srep29774)

Chloroquine enhanced the anticancer capacity of VNP20009 by inhibiting autophagy

Xiaoxin Zhang^1^, Qiaoqiao Xu^1^, Zhuangzhuang Zhang^1^, Wei Cheng^1^, Wenmin Cao^1^, Chizhou Jiang^1^, Chao Han^1^, Jiahuang Li^1,2^, Zichun Hua^1,3,4,*^

^1^The State Key Laboratory of Pharmaceutical Biotechnology, School of Life Science and School of Stomatology, Affiliated Stomatological Hospital, Nanjing University, Nanjing, 210093, Jiangsu, China.

^2^Changzhou High-Tech Research Institute of Nanjing University and Targetpharma Laboratory, Changzhou 213164, Jiangsu, China.

^3^College of Pharmacy, Nanjing University of Chinese Medicine, Nanjing 210046, China.

^4^The State Key Laboratory of Bioelectronics, Southeast University, Nanjing 210018, China.

^*^Corresponding author: Zi-Chun Hua

E-mail: zchua@nju.edu.cn.

**Supplementary Fig. S1**

**VNP20009 induced autophagy in human A375 melanoma cells.** (a) A375 cells transfected with GFP-LC3 were infected with VNP20009 and fixed at indicated times post transfection. Cells were then stained with DAPI to determine nucleus. Cells were analyzed by fluorescence microscopy. (b) The formation of vacuoles containing GFP-LC3 per cell was analyzed by Image-Pro Plus. Data are presented as mean ± SD. **, p＜0.01, as compared with untreated group. (c) A375 cells were infected as in A. Immunoblot analyses for LC3-II.

**
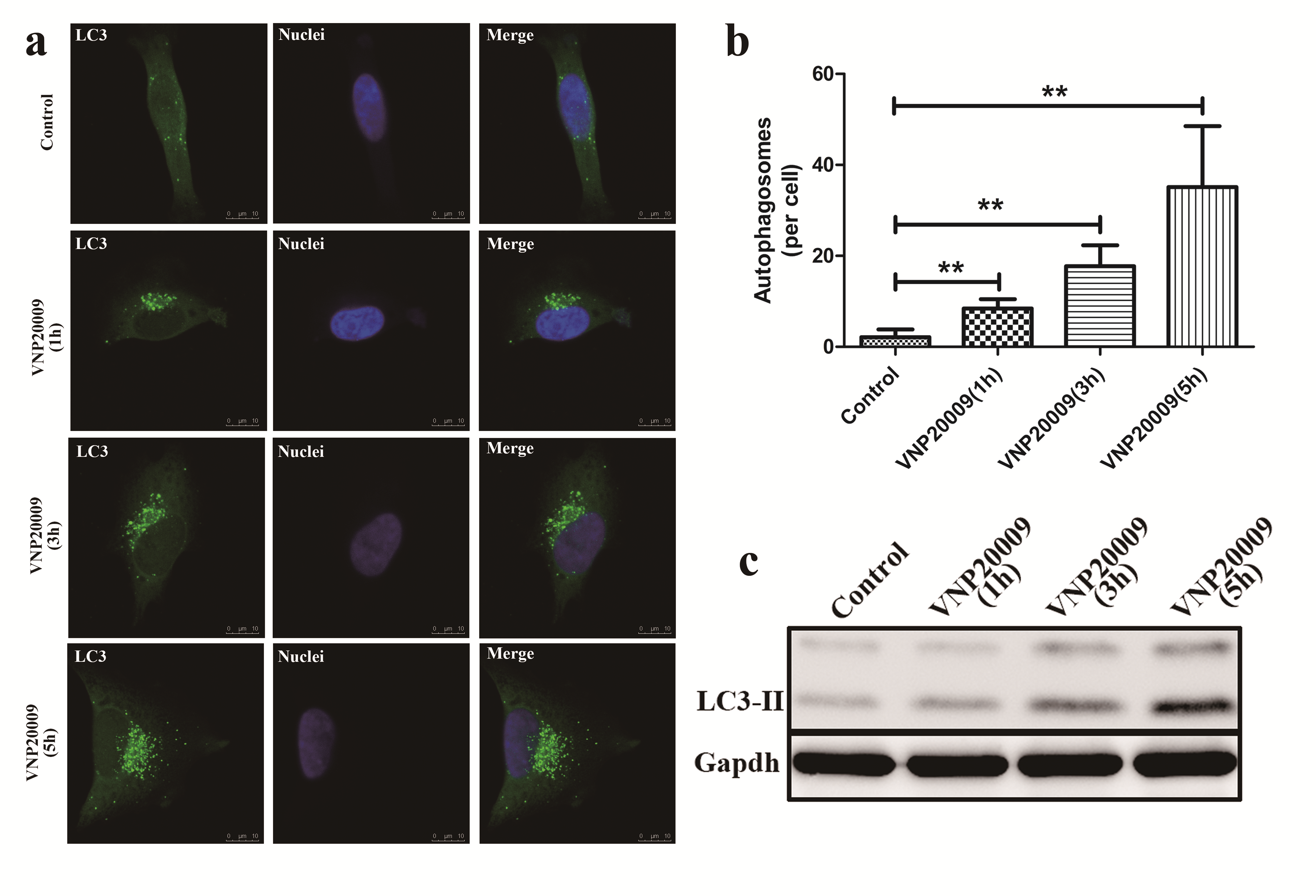
**

**Supplementary Fig. S2**

**Inhibition of autophagy by siRNA-ATG7 induced more cell death than VNP20009 treatment**. (a) ATG7 relative expression by qPCR from B16F10 lysates. Data are presented as mean ± SD. *, p＜0.01 (b) Protein expression of LC3-II after infected with VNP20009 in the wild type or ATG7 knock out cells. (c) The wild type or ATG7 knock out cells were infected with VNP20009 at different MOI. Cell death was analyzed using flow cytometry. Data are presented as mean ± SD. **, p＜0.01. (d) The wild type or ATG7 knock out cells were infected with VNP20009 at MOI of 10:1 at indicated time. Cell death was analyzed using flow cytometry. Data are presented as mean ± SD. **, p＜0.01.


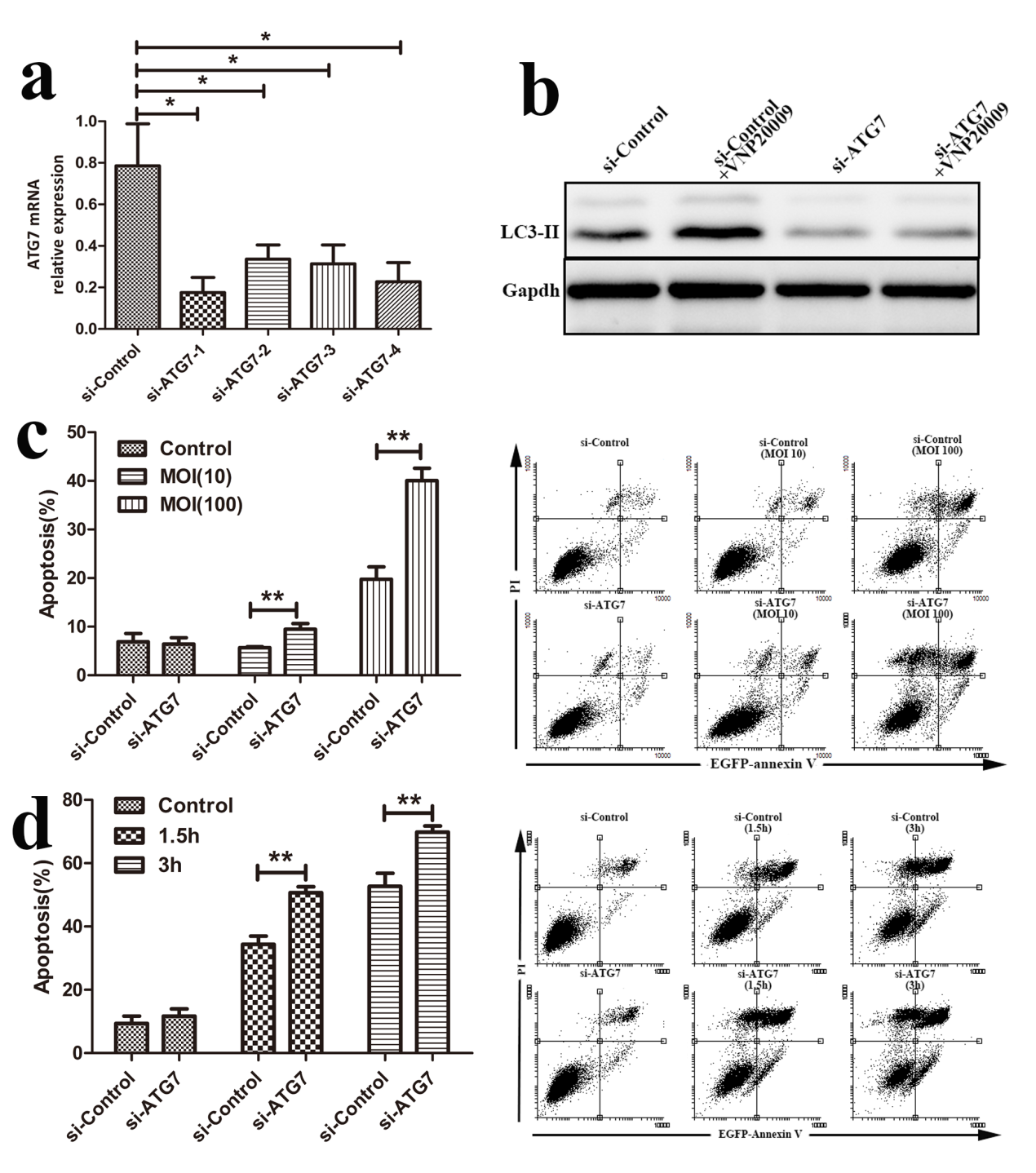

Supplement: Supplementary Information [file srep29774-s1.doc]
